# Supplementary material for: Potentially Asymptomatic Infection of Monkeypox Virus: A Systematic Review and Meta-Analysis
Source: Vaccines (Basel). 2022 Dec 6;10(12):2083. doi: 10.3390/vaccines10122083 (PMC9784491; doi:10.3390/vaccines10122083)
Supplement: Supplementary file 1 [file vaccines-10-02083-s001.zip › vaccines-2042139-supplementary.pdf]

Manuscript Title: Potential Asymptomatic Infection of Monkeypox Virus: A Systematic Review and Meta-Analysis

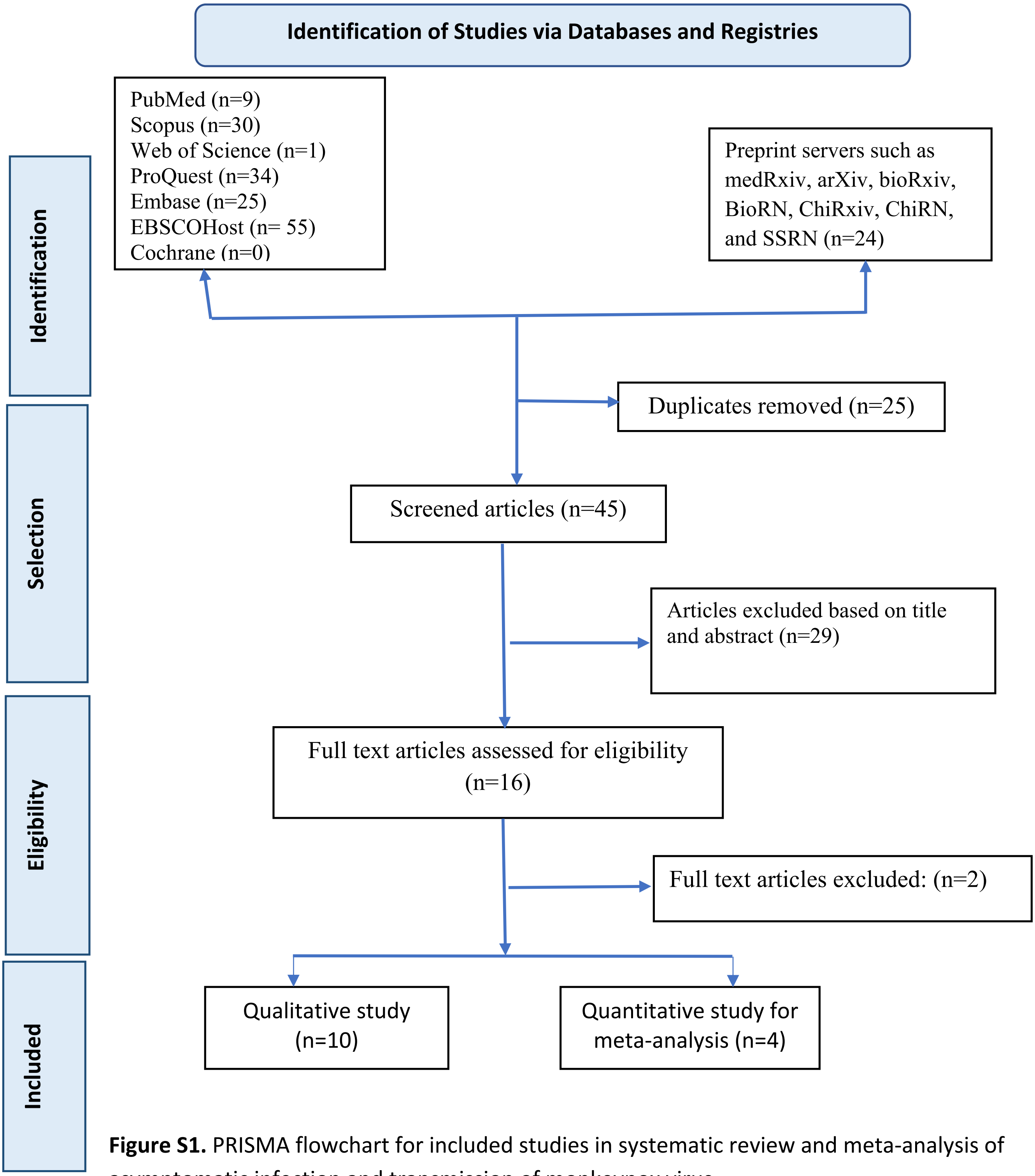

Figure S1. PRISMA flowchart for included studies in systematic review and meta-analysis of asymptomatic infection and transmission of monkeypox virus

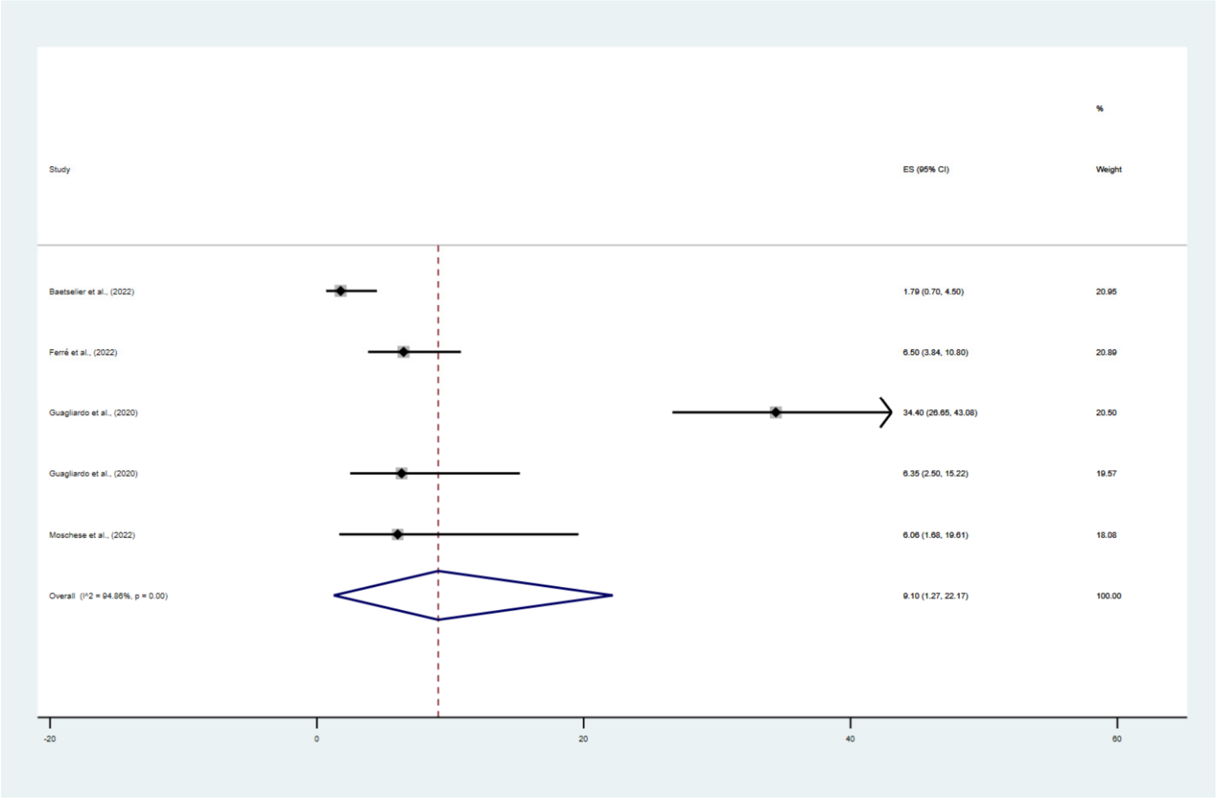

Figure S2. Forest plot of pooled magnitude of asymptomatic monkeypox

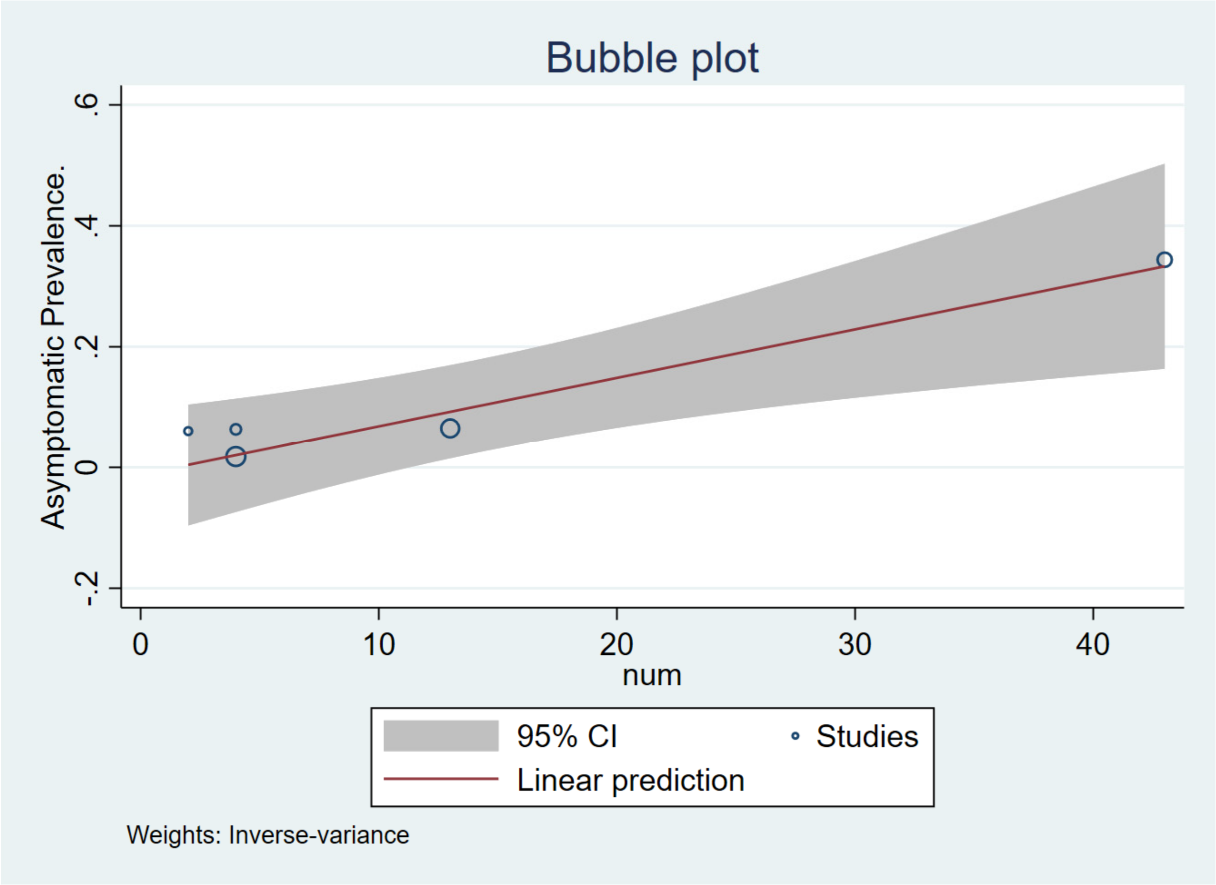

Figure S3. Bubble plot with 95% Confidence interval of pooled prevalence of asymptomatic monkeypox
